# Supplementary material for: Low-Dose Aspirin Use Significantly Improves the Survival of Late-Stage NPC: A Propensity Score-Matched Cohort Study in Taiwan
Source: Cancers (Basel). 2020 Jun 12;12(6):1551. doi: 10.3390/cancers12061551 (PMC7352863; doi:10.3390/cancers12061551)
Supplement: Supplementary file 1 [file cancers-12-01551-s001.pdf]

cancers

MDPI

Supplementary Materials

Low-dose Aspirin Use Significantly Improves the Survival of Late-stage NPC: A Propensity Score-Matched Cohort Study in Taiwan

Sheng-Dean Luo, Wei-Chih Chen, Ching-Nung Wu, Yao-Hsu Yang, Shau-Hsuan Li, Fu-Min Fang, Tai-Lin Huang, Yu-Ming Wang, Tai-Jan Chiu and Shao-Chun Wu

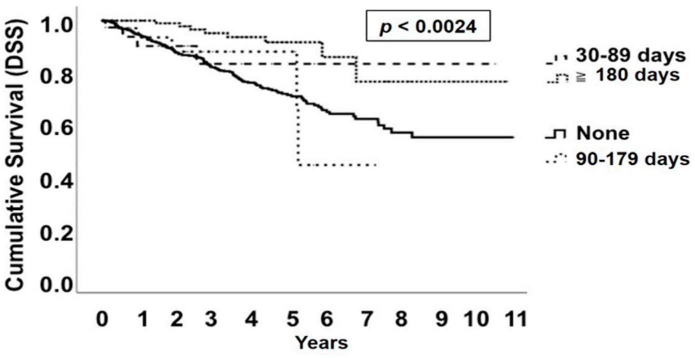

Variable	Cohort n = 565	Survival Rates (%) Years										*p*-value
		1	2	3	4	5	6	7	8	9	10	
Aspirin Use												
30-89 days	38(4.0%)	90.2	90.2	83.2	83.2	83.2	83.2	83.2	83.2	83.2	83.2	<0.0024**
90-179 days	40(4.2%)	93.5	88.0	88.0	88.0	88.0	44.0	44.0	-	-	-	
≧180 days	113(11.8%)	100.0	99.0	95.1	93.6	91.6	85.9	76.3	76.3	76.3	76.3	
None	764(80.0%)	94.6	87.6	81.9	75.9	71.1	64.6	61.9	56.6	54.7	54.7	

Figure S1. Kaplan-Meier survival curve of disease-specific survival (DSS) among 30–89-day, 90–179-day, ≥ 180-day aspirin users and non-users. Kaplan-Meier survival curves of 30–89-day aspirin users (n = 38), 90–179-day aspirin users (n = 40), ≥ 180-day aspirin users (n = 113) and non-users (n = 764). A total of 2666 patients diagnosed with NPC were recruited for this study. Aspirin users were matched with non-users (None) based on a 1:4 propensity score, resulting in a final inclusion of 955 patients with NPC for data analysis. The estimated 5- and 10-year DSS rates of 30–89-day aspirin users were both 83.2%. The estimated 5-year DSS rate of 90–179-day aspirin users was 88.0%. The estimated 5- and 10-year DSS rates of ≥ 180-day aspirin users were 91.6% and 76.3%, respectively. The estimated 5- and 10-year DSS rates of aspirin non-users (None) were 71.1% and 54.7%, respectively. p-value was 0.0024 for the overall comparison among the groups using the log rank test. p-values of pairwise comparisons were as follows: non-users versus 30–89-day aspirin users was 0.4087; non-users versus 90–179-day aspirin users was 0.9082; non-user versus ≥ 180-day aspirin users was 0.0002; 30–89-day aspirin user versus 90–179-day aspirin users was 0.6401; 30–89-day aspirin users versus ≥ 180-day aspirin users was 0.1613; 90–179-day aspirin users versus ≥ 180-day aspirin users was 0.0118.

Table S1. Baseline characteristics of NPC patients before and after propensity-score matching.

Characteristics	Before Propensity Score Matching			After Propensity Score Matching		
	Non-users (Control) *n* = 2090	Aspirin Users ≥ 180 days (Intervention) *n* = 113	Standardized Mean Difference	Non-users (Control) *n* = 452	Aspirin Users ≥ 80 days (Intervention) *n* = 113	Standardized Mean Difference
**Propensity Score**	0.06 ± 0.05	0.10 ± 0.05	0.7917	0.10 ± 0.05	0.10 ± 0.05	0.0196
**Sex**						
Male (%)	74.4	82.3	0.1934	83.8	82.3	0.0412
Female (%)	25.6	17.7	0.1934	16.2	17.7	0.0412
**Age**						
≤ 60 years(%)	82.3	70.8	0.2745	75.2	70.8	0.0995
> 60 years(%)	17.7	29.2	0.2745	24.8	29.2	0.0995
**Stages of Cancer (AJCC)**[a]						
I & II (%)	28.6	42.5	0.2931	40.5	42.5	0.0403
III & IV[b] (%)	71.4	57.5	0.2931	59.5	57.5	0.0403

[a] AJCC Cancer Staging 7th Edition.

Generally, one-to-many or many-to-one matching is employed in observational studies that have a greater number of untreated patients than treated patients [1–5]. It has been shown that a matching ratio no greater than 1:4 (treated patients to untreated patients) may increase precision while generate the least biased estimates for treatment effect [1–5]. In addition, further matching with a caliper width of 0.25 standard deviations provides a better balance between treated and untreated patients, which was also used by other studies as well [6,7]. Considering to achieve a balanced covariate distribution between treated and untreated patients, therefore, we used propensity score Mahalanobis 1:4 matching with a caliper width of 0.25 standard deviations to match non-users (452) to each aspirin-users (113) based on covariates listed in Table S1. The standardized mean difference (SMD) of the dichotomous categorical covariate should be less than 0.1 (closer to zero is better) in order to be considered as having a good balance of covariate distribution between users and non-users [5]. Before the propensity-score matching, all of the covariates listed in Table S1 had SMDs greater than 0.1, suggesting that there were imbalances in covariates between aspirin users and non-users. The imbalances in covariates between aspirin users and non-users were resolved after applying propensity score 1:4 matching, in which all of the covariates listed in Table S1 had SMDs lesser than 0.1.

Table S2. Univariate and multivariate Cox proportional hazards models of prognostic factors for NPC survival (additionally adjusted for comorbidities)

Variables	Cohort *n* = 565 *n* (%)	Hazard Ratio (95% CI)			
		Univariate	*p*-value	Multivariate	*p*-value
**Sex**					
Female	93 (16.5)	1	0.372	1	0.815
Male	472 (83.5)	1.26 (0.76–2.11)		1.07 (0.63–1.82)	
**Age**					
≤ 60 years	420 (74.3)	1	< 0.001 ***	1	< 0.001 ***
> 60 years	145 (25.7)	2.05 (1.43–2.95)		2.13 (1.43–3.16)	
**Stages of Cancer (AJCC)**[a]					
I & II	231 (40.9)	1	< 0.001 ***	1	< 0.001 ***
III & IV[b]	334 (59.1)	3.88 (2.45–6.15)		3.65 (2.91–6.08)	
**Treatments**					
CCRT	470 (83.2)	1		1	
RT	80 (14.2)	0.78 (0.44–1.39)	0.405[c]	1.09 (0.58–2.04)	0.786[e]
CT	9 (1.5)	29.63 (12.14–72.34)	< 0.001 *** [d]	35.42 (13.40–93.60)	< 0.001 ***[f]
**Aspirin Use**					
No	113 (20.0)	1	< 0.001 ***	1	0.022 *
≥ 180 days	145 (25.7)	0.28(0.14–0.55)		0.39 (0.18–0.88)	
**Comorbidities**					
CVA					
No	451 (79.8%)	1	< 0.001 ***	1	0.003 **
Yes	114 (20.2%)	0.03 (0.004–0.20)		0.05 (0.01–0.36)	
DM					
No	490(86.7%)	1	0.746	1	0.342
Yes	75(13.3%)	0.92 (0.54–1.55)		1.31 (0.75–2.31)	
Hypertension					
No	434 (76.8%)	1	0.129	1	0.373
Yes	131 (23.2%)	0.70 (0.45–1.11)		0.78 (0.46–1.34)	
Atrial fibrillation (flutter)					
No	559 (98.8%)	1	0.068	1	0.009 *
Yes	6 (1.1%)	2.91 (0.93–9.18)		5.13 (1.51–17.41)	
Hyperlipidemia					
No	389 (68.8%)	1	0.001 **	1	0.803
Yes	176 (31.2%)	0.46 (0.30–0.72)		0.94 (0.56–1.56)	

Abbreviations: 95% CI 95% confidence interval; CCRT concurrent chemoradiotherapy; RT radiotherapy; CT chemotherapy; CVA cerebrovascular accident; DM diabetes mellitus; [a] AJCC Cancer Staging 7th Edition; [b] Stages IVa and IVb only; [c, e] Comparing RT to CCRT; [d, f] Comparing CT to CCRT. * $p \leq 0.05$; ** $p \leq 0.01$; *** $p \leq 0.001$.

We evaluated the univariate and multivariate analyses of independent prognostic factors for survival with additional adjustment for the five comorbidities, including CVA, DM, hypertension, atrial fibrillation and hyperlipidemia. In the univariate Cox regression analysis, various clinical variables, including age ($HR_{\leq 60 \text{ vs. } >60}$ = 2.05, 95% CI = 1.43–2.95, $p < 0.001$), AJCC stages of cancer ($HR_{\text{stages I and II vs. stages III and IV}}$ = 3.88, 95% CI = 2.45–6.15, $p < 0.001$), CT ($HR_{\text{CT treatment vs. CCRT standard NPC treatment}}$ = 29.63, 95% CI = 12.14–72.34, $p < 0.001$), and aspirin use ($HR_{\text{aspirin non-users vs. users}}$ = 0.28, 95% CI = 0.14–0.55, $p < 0.001$), CVA ($HR_{\text{CVA No vs. Yes}}$ = 0.03, 95% CI = 0.004–0.20, $p < 0.001$), and hyperlipidemia ($HR_{\text{hyperlipidemia No vs. Yes}}$ = 0.46, 95% CI = 0.30–0.72, $p < 0.001$) were significantly associated with the survival rate, while patient sex ($HR_{\text{females vs. males}}$ = 1.26, 95% CI = 0.76–2.11, p = 0.372) and RT ($HR_{\text{RT treatment vs. CCRT standard NPC treatment}}$ = 0.78, 95% CI = 0.44–1.39, p = 0.405), DM ($HR_{\text{DM No vs. Yes}}$ = 0.92, 95% CI = 0.54–1.55, p = 0.746), hypertension ($HR_{\text{hypertension No vs. Yes}}$ = 0.70, 95% CI = 0.45–1.11, p = 0.129), atrial fibrillation ($HR_{\text{atrial fibrillation No vs. Yes}}$ = 2.91, 95% CI = 0.93–9.18, p = 0.068) were not significantly related to survival. The multivariate Cox regression analysis showed that various clinical variables, including age ($HR_{\leq 60 \text{ vs. } >60}$ = 2.13, 95% CI = 1.43–3.16, $p < 0.001$), AJCC stages of cancer ($HR_{\text{stages I and II vs. stages III and IV}}$ = 3.65, 95% CI = 2.91–6.08, $p < 0.001$), CT ($HR_{\text{CT treatment vs. CCRT standard NPC treatment}}$ = 35.42, 95% CI = 13.40–93.60, $p < 0.001$), and aspirin use ($HR_{\text{aspirin non-users vs. users}}$ = 0.39, 95% CI = 0.18–0.88, p = 0.022), and CVA ($HR_{\text{CVA No vs. Yes}}$ = 0.05, 95% CI = 0.01–0.36, p = 0.003) were significantly associated with survival. In contrast, patient sex ($HR_{\text{females vs. males}}$ = 1.07, 95% CI = 0.63–1.82, p = 0.815), RT ($HR_{\text{RT treatment vs. CCRT standard NPC treatment}}$ = 1.29, 95% CI = 0.67–2.48, p = 0.454),

and DM ($HR_{DM\ No\ vs.\ Yes}$ = 0.92, 95% CI = 0.54–1.55, p = 0.746, HRs) were not independent prognostic factors for survival. Variable such as atrial fibrillation was not significant in univariate analysis but became significant in multivariate analysis ($HR_{atrial\ fibrillation\ No\ vs.\ Yes}$ = 5.13, 95% CI = 1.51–17.41, p = 0.009). Unlike atrial fibrillation, the univariate analysis of hyperlipidemia was significant but ($HR_{hyperlipidemia\ No\ vs.\ Yes}$ = 0.94, 95% CI = 0.56–1.56, p = 0.803) did not reach statistical significant in multivariate analysis.

Table S3. Cox proportional hazards models of aspirin use for NPC survival.

Models	Hazard Ratio	(95% CI)	*p*-value
Univariate	0.28	0.14–0.55	< 0.001 ***
Main Model [a] (Table 2)	0.23	0.12–0.46	< 0.001 ***
Main Model + adjusted for CVA	0.40	0.20–0.81	< 0.011 *
Main Model + adjusted for DM	0.23	0.11–0.45	< 0.001 ***
Main Model + adjusted for Hypertension	0.25	0.13–0.51	< 0.001 ***
Main Model + adjusted for Atrial fibrillation (flutter)	0.19	0.09–0.40	< 0.001 ***
Main Model + adjusted for Hyperlipidemia	0.27	0.13–0.56	< 0.001 ***
Main Model + adjusted for All 5[b] comorbidities	0.39	0.18–0.88	0.022 *

Abbreviations: 95% CI 95% confidence interval; CVA cerebrovascular accident; DM diabetes mellitus; [a] Adjusted for sex, age, stages of cancer (AJCC), treatments, aspirin use; [b] CVA, DM, hypertension, atrial fibrillation (flutter), hyperlipidemia. * $p \leq 0.05$; *** $p \leq 0.001$.

Various Cox regression models were used to examine the associations between aspirin use and NPC cancer survival. Although the associations between aspirin use and NPC cancer survival were either slightly attenuated or enhanced in these different models, aspirin use is still displayed as a good prognostic factor for NPC survival.

Table S4. Univariate and multivariate Cox proportional hazards models of prognostic factors for survival of NPC patients with CCRT treatment (n = 470).

Variables	Cohort (CCRT) *n* = 470	Hazard Ratio (95% CI)			
		Univariate	*p*-value	Multivariate	*p*-value
**Sex**					
Female	74 (15.7%)	1	0.836	1	0.810
Male	472 (83.5%)	0.95 (0.56–1.61)		0.94 (0.55–1.61)	
**Age**					
≤ 60 years	361 (76.8%)	1	< 0.001 ***	1	< 0.001 ***
> 60 years	109 (23.2%)	2.15 (1.44–3.22)		2.07 (1.38–3.11)	
**Stages of Cancer (AJCC)**[a]					
I & II	167 (35.5%)	1	< 0.001 ***	1	< 0.001 ***
III & IV[b]	303 (64.5%)	3.54 (2.08–6.03)		3.43 (2.01–5.85)	
**Aspirin Use**					
No	365 (77.7%)	1	< 0.001 ***	1	< 0.001 ***
≥ 180 days	105 (22.3%)	0.30 (0.15–0.59)		0.27 (0.14–0.54)	

Abbreviations: 95% CI 95% confidence interval; CCRT concurrent chemoradiotherapy; [a] AJCC Cancer Staging 7th Edition; [b] Stages IVa and IVb only. *** $p \leq 0.001$.

The univariate Cox regression analysis showed that various clinical variables, including age ($HR_{\leq 60\ vs.\ >60}$ = 2.15, 95% CI = 1.44–3.22, $p < 0.001$), AJCC stages of cancer ($HR_{stages\ I\ and\ II\ vs.\ stages\ III\ and\ IV}$ = 3.54, 95% CI = 2.08–6.03, $p < 0.001$) and aspirin use ($HR_{aspirin\ non\text{-}users\ vs.\ users}$ = 0.30, 95% CI = 0.15–0.59, $p < 0.001$) were significantly associated with the survival rate, while patient sex ($HR_{females\ vs.\ males}$ = 0.95, 95% CI = 0.56–1.61, p = 0.836) was not significantly related to survival. The multivariate Cox regression analysis, the results of which were similar to those of the univariate analysis, indicated that the factors of age ($HR_{\leq 60\ vs.\ >60}$ = 2.07, 95% CI = 1.38–3.11, $p < 0.001$), AJCC stages of cancer ($HR_{stages\ I\ and\ II\ vs.\ stages\ III\ and\ IV}$ = 3.43, 95% CI = 2.01–5.85, $p < 0.001$), and aspirin use ($HR_{aspirin\ non\text{-}users\ vs.\ users}$ = 0.27, 95% CI = 0.14–0.54, $p < 0.001$) were significantly associated with survival, whereas patient sex ($HR_{females\ vs.\ males}$ = 0.94, 95% CI = 0.55–1.61, p = 0.810) was not an independent prognostic factor for survival (Table S4).

CCRT is the standard cancer treatment for NPC patients. Most of the NPC patients are treated with CCRT treatment after diagnosis of NPC. Considering CCRT may have an effect on the

association between aspirin use and NPC cancer survival, therefore, we addressed this question by running the univariate and multivariate Cox regression analyses with NPC patients who only treated with CCRT. Both stages I and II and III and IV NPC patients had CCRT treatment in this statistical analysis. In this case, having stages III and IV of NPC increased the hazard by a factor of 3.43 as compared to patients with stages I and II of NPC who were also treated with CCRT. Therefore, having stages III and IV of NPC was associated with bad prognostic even though these patients were treated with CCRT as patients with stages I and II of NPC. Similarly, patients had CCRT treatment regardless whether they were aspirin users or not, having aspirin reduced the hazard by a factor of 0.27 as compared to patients who treated with CCRT but without aspirin use. Therefore, our results demonstrated that having aspirin is associated with good prognostic, suggesting that a protective association for survival in NPC patients who were treated with CCRT is due to aspirin use.

References

1. Linden, A.; Samuels, S.J. Using balance statistics to determine the optimal number of controls in matching studies. *J. Evaluat. Clin. Pr.* **2013**, *19*, doi:10.1111/jep.12072.
2. Abadie, A.; Imbens, G. Simple and Bias-Corrected Matching Estimators for Average Treatment Effects. *Nat. Bureau Econ. Res.* **2002**, doi:10.3386/t0283.
3. Austin, P.C. Statistical criteria for selecting the optimal number of untreated subjects matched to each treated subject when using many-to-one matching on the propensity score. *Am. J. Epidemiol.* **2010**, *172*, 1092–1097, doi:10.1093/aje/kwq224.
4. Smith, H.L. 6. Matching with Multiple Controls to Estimate Treatment Effects in Observational Studies. *Sociol. Methodol.* **1997**, *27*, 325–353, doi:10.1111/1467-9531.271030.
5. Jacovidis, J.N. Evaluating the Performance of Propensity Score Matching Methods: A Simulation Study; Doctoral Dissertation, James Madison University: Virginia, USA, 2017.
6. Lunt, M. Selecting an appropriate caliper can be essential for achieving good balance with propensity score matching. *Am. J. Epidemiol.* **2013**, *179*, 226–235, doi:10.1093/aje/kwt212.
7. Cochran, W.G.; Rubin, D.B. Controlling Bias in Observational Studies: A Review. *Match. Sampl. Causal Eff.* **2012**, 30–58, doi:10.1017/cbo9780511810725.005.

© 2020 by the authors. Licensee MDPI, Basel, Switzerland. This article is an open access article distributed under the terms and conditions of the Creative Commons Attribution (CC BY) license (http://creativecommons.org/licenses/by/4.0/).
